# *Ageratum enation virus* Infection Induces Programmed Cell Death and Alters Metabolite Biosynthesis in *Papaver somniferum*

**DOI:** 10.3389/fpls.2017.01172

**Published:** 2017-07-06

**Authors:** Ashish Srivastava, Lalit Agrawal, Rashmi Raj, Meraj Jaidi, Shri K. Raj, Swati Gupta, Ritu Dixit, Poonam C. Singh, Tusha Tripathi, Om P. Sidhu, Brahma N. Singh, Sudhir Shukla, Puneet S. Chauhan, Susheel Kumar

**Affiliations:** ^1^Plant Molecular Virology Laboratory, Council of Scientific and Industrial Research – National Botanical Research InstituteLucknow, India; ^2^Amity Institute of Virology and Immunology, Amity UniversityNoida, India; ^3^Division of Plant Microbe Interaction, Council of Scientific and Industrial Research – National Botanical Research InstituteLucknow, India; ^4^Division of Phytochemistry, Council of Scientific and Industrial Research – National Botanical Research InstituteLucknow, India; ^5^Division of Pharmacognosy and Ethnopharmacology, Council of Scientific and Industrial Research – National Botanical Research InstituteLucknow, India; ^6^Plant Breeding Laboratory, Council of Scientific and Industrial Research – National Botanical Research InstituteLucknow, India

**Keywords:** begomovirus, biotic stress, defense enzymes, HPLC, GC-MS, opium poppy

## Abstract

A previously unknown disease which causes severe vein thickening and inward leaf curl was observed in a number of opium poppy (*Papaver somniferum* L.) plants. The sequence analysis of full-length viral genome and associated betasatellite reveals the occurrence of *Ageratum enation virus* (AEV) and Ageratum leaf curl betasatellite (ALCB), respectively. Co-infiltration of cloned agroinfectious DNAs of AEV and ALCB induces the leaf curl and vein thickening symptoms as were observed naturally. Infectivity assay confirmed this complex as the cause of disease and also satisfied the Koch’s postulates. Comprehensive microscopic analysis of infiltrated plants reveals severe structural anomalies in leaf and stem tissues represented by unorganized cell architecture and vascular bundles. Moreover, the characteristic blebs and membranous vesicles formed due to the virus-induced disintegration of the plasma membrane and intracellular organelles were also present. An accelerated nuclear DNA fragmentation was observed by Comet assay and confirmed by TUNEL and Hoechst dye staining assays suggesting virus-induced programmed cell death. Virus-infection altered the biosynthesis of several important metabolites. The biosynthesis potential of morphine, thebaine, codeine, and papaverine alkaloids reduced significantly in infected plants except for noscapine whose biosynthesis was comparatively enhanced. The expression analysis of corresponding alkaloid pathway genes by real time-PCR corroborated well with the results of HPLC analysis for alkaloid perturbations. The changes in the metabolite and alkaloid contents affect the commercial value of the poppy plants.

## Introduction

Geminiviruses of the family *Geminiviridae* infect a large range of crop and non-crop plants, and are considered as a major threat to the agriculture system globally, but consequences are more severe in warmer parts of the world. With the identification of novel viruses, the family *Geminiviridae* is extended into nine genera namely *Mastrevirus*, *Curtovirus*, *Topocuvirus*, *Becurtovirus*, *Eragrovirus*, *Capulavirus*, *Grablovirus*, *Begomovirus*, and *Turncurtovirus*, depending on the host range, insect-vector and genome characteristics (Zerbini et al., 2017). Amongst the known, whitefly (*Bemisia tabaci* Genn.) transmitted begomoviruses are the largest plant infecting DNA virus genera (Brown et al., 2015). Begomoviruses have single-stranded circular DNA genome of about 2.8 kb coding for 5–7 viral proteins (Hanley-Bowdoin et al., 2013). These proteins collectively take over the host cellular processes, interfere with and suppress the plant defense system causing infection, and as a consequence the plants develop symptoms like leaf curling, leaf yellowing, leaf enation, leaf crumpling, vein thickening, and plant stunting. Begomoviruses either have a monopartite genomic organization and possess a DNA-A like genome which is mostly associated with satellites (alphasatellite and/or betasatellite) or a bipartite genomic organization with two circular DNA components, designated as DNA-A and DNA-B (Zhou, 2013). Begomoviruses are the fastest co-evolving plant viruses and cause significant economic losses (Jeske, 2009). Besides infecting the food, fiber, and weed plants (Castillo-Urquiza et al., 2008), they have also been reported to infect the medicinal and aromatic plants and affect produce quality (Saeed and Samad, 2016).

Plants produce primary and secondary metabolites of varied structures. The crop yield and the quality of produce depend on the quantity of metabolites produced by the plant. Metabolites play an important role in plant development and respond to diverse environmental conditions (Scossa et al., 2016). Begomovirus infection in host plants triggers an array of morphological, biochemical and molecular changes (Mandadi and Scholthof, 2013). In *Amaranthus hypochondriacus*, infection by *Ageratum enation virus* (AEV) has been reported as the cause of alteration in sap translocation, and variation in primary and secondary metabolites, besides disfiguring the cell structures (Srivastava et al., 2012). Similarly, alterations in the cell structures, phloem tissue, and metabolites were also observed in *Jatropha curcas* plants infected by *Jatropha mosaic virus* (Sidhu et al., 2010). In cotton, deterioration in fiber quality traits like ginning out turn percentage, staple length, fiber uniformity index, fiber fineness, fiber bundle strength, and maturity ratio was observed due to the infection by *Cotton leaf curl begomovirus* (Farooq et al., 2011). To counteract the virus infection, plants have the ability to recognize the invading pathogen and respond against them by a variety of defense systems (Bellincampi et al., 2014). The defense systems include synthesizing toxic metabolites and pathogen-degrading enzymes, and apoptosis, an induced programmed cell death (PCD) (Moshe et al., 2016). PCD plays important roles in a number of plant developmental pathways including xylogenesis, formation of woody tissues, leaf abscission, and formation of laticifers. It’s pronounced role in the defense response against a wide variety of plant pathogens and environmental stresses was also reported (Eckardt, 2006). A monopartite begomovirus *Cabbage leaf curl virus* (CaLCuV) induced PCD together with genotoxic response and cell cycle changes was observed in agroinfiltrated *Arabidopsis* plants (Ascencio-Ibanez et al., 2008). Further, real time-PCR analysis of the genes coding for proteins involved in PCD suggested the gene expression alteration induced by CaLCuV. Bass et al. (2000) revealed the chromatin condensation induced PCD in the leaf cells of *Nicotiana benthamiana* plants infiltrated with agroinfectious cloned DNAs of *Tomato golden mosaic virus* (TGMV). These examples suggest that begomoviruses have propensity to accelerate PCD in plants.

Opium poppy (*Papaver somniferum* L., family Papaveraceae, *2n = 22*) is one of the oldest medicinal herbs known to mankind and is cultivated on a large scale in India, Iran, Turkey, Holland, Poland, Romania, Czechoslovakia, Yugoslavia, Canada, and many Central and South American countries for opium content^1^. Owing to its potent biological activity, about 1200 known alkaloids have been exploited as pharmaceuticals, stimulants, narcotics, and poisons (Zaim et al., 2014). The pharmacologically active benzylisoquinoline alkaloids are represented by two main groups: the phenanthrenes contain the narcotic analgesics morphine, codeine, and thebaine; and isoquinolines contain the muscle relaxant and vasodilator papaverine and the antineoplastic drug, noscapine (Desgagné-Penix and Facchini, 2012). Poppy is mainly reported to be infected by *Peronospora* species of fungus causing the downy mildew disease (Voglmayr et al., 2014). Moreover, poppy plants were also known as host to several RNA viruses like *Bean yellow mosaic virus*, *Turnip mosaic virus*, *Poppy mosaic virus*, and *Beet mosaic virus* (Pethybridge et al., 2005; Zaim et al., 2014). Recently, we have identified a bipartite begomovirus, *Tomato leaf curl New Delhi virus* (ToLCNDV), associated with leaf curl disease in opium poppy without much impact on crop yield (Srivastava et al., 2016). In further surveys, the opium poppy plants showed much severe disease symptoms of inward leaf curl coupled with severe vein thickening. We identified for the first time, a monopartite begomovirus which infects these plants, which encourages us to do the comprehensive histological, biochemical, and metabolomic investigations. Further, the data is well supported by the molecular analysis to assess the viral disease impact on infected plants, and to delineate the unaddressed post-viral infection consequences.

## Materials and Methods

### Plant Materials and Virus Detection

Infected *P. somniferum* plants exhibiting severe disease symptoms were evaluated at experimental breeding plots of CSIR-NBRI, India (Latitude 26° 51′N Longitude 80° 55′E). For virus identification and estimation of plant morphological parameters, 10 randomly selected infected plant samples along with a healthy sample (as control) were collected.

For virus detection, total DNA and RNA was independently isolated from plant samples using GenElute Plant Genomic DNA isolation (Sigma–Aldrich, United States) and Spectrum Plant Total RNA isolation (Sigma–Aldrich, United States) kits following manufacturers’ instructions. For RNA and DNA virus detection, the reverse transcription (RT)-PCR and PCR were performed using the respective degenerate primers (Supplementary Table S1). All PCR products were separated by electrophoresis on 1% agarose gel and corresponding band size was assessed using 1.0 kb DNA ladder (Thermo Fisher Scientific Inc., United States).

### Full-Length Genome Amplification, Virus Identification and Transient Expression of Viral DNAs

The full-length genome of begomovirus was amplified from a plant DNA sample using unbiased rolling circle amplification (RCA) method (Illustra TempliPhi Amplification Kit, GE Healthcare Life Sciences, United States). The concatemer of RCA product was made into monomers by restriction digestion with *Bam*HI enzyme. The DNA fragment of ∼2.8 kb so obtained was gel purified and cloned at the *Bam*HI site in pCAMBIA1300 vector. Several chimeric clones were screened by restriction digestion with *Bam*HI for DNA insert, and a positive clone was sequenced by primer walking (Genei Pvt. Ltd., India). The virus associated satellite DNA amplified from the same symptomatic plant sample was cloned in pGEM-T easy vector system-I (Promega Corp., United States) and sequenced. Viral sequences were assembled, analyzed to eliminate the sequence ambiguity, and deposited in the NCBI GenBank database (Accession numbers JQ911765 and KJ948106). The open reading frames (ORFs) within the sequences were predicted by an ORF finder^2^ to find out in the frame AUG (ATG)-start and UAG (TAG)-termination codons. To assess the phylogenetic relationship of virus under study, trees were constructed employing MEGA v6.1 tool using the Maximum Composite Likelihood method with 1000 bootstrap replicates. The percentage of trees in which the associated taxa clustered together is shown next to the branches. The trees are drawn to scale with branch lengths measured in the number of substitutions per site. All positions containing gaps and missing data were eliminated.

To determine the infectivity, agroinfectious clones of AEV and ALCB, DNAs for transient expression were developed following the conditions described earlier (Srivastava et al., 2013). The syringe infiltration method was used to infiltrate 4–6 leaf stage *P. somniferum*, *Nicotiana glutinosa*, *Solanum lycopersicon*, and *Ageratum conyzoides* test plants. The infiltrated plants were kept in glasshouse conditions (natural illumination with temperature of 25 ± 2°C) and examined regularly for the appearance of symptoms.

### Assessment of Plant Morphological and Biochemical Parameters

The morphological parameters such as shoot length (cm), shoot diameter (cm) (immediately after node of 6th leaf which is in the middle of the plant), diameter of capsule (cm), size of top third leaf (cm), fresh and dry weight (g) of 10 infected and 10 healthy opium poppy plants were recorded at the time of sample collection from 60-day post-germinated plants. The estimation of total chlorophyll, proline and lipid peroxidation was done in both infected and healthy plants as given in Supplementary Method S1. The activity of antioxidant enzymes like superoxide dismutase (SOD), catalase (CAT), ascorbate peroxidase (APX) was also measured using the crude enzyme extract as given in Supplementary Method S1.

### Histology of Virus Infected and Healthy Opium Poppy Plant

#### Anatomical Study by Light Microscopy

For anatomical studies (changes in tissue profiles), hand-cut sections of the stem and leaves of infected and healthy poppy plants were examined. Samples were fixed and rehydrated in FAA (formaldehyde:acetic acid:alcohol::5:5:90, v/v) for a week and then preserved in alcohol-glycerol mixture (1:1 mixture of 70% ethyl alcohol and glycerol). The washed samples were hand sectioned using fresh blades. The sections were dehydrated in a graded ethanol series and differentially stained using fast green and safranin. The red stained cells represent dead cells while blue to green stained cells represent alive cells.

#### Ultrastructural Investigation by Scanning Electron Microscope (SEM)

The leaf surface of naturally infected and healthy poppy plants was studied by high resolution field emission E.M., Quanta SEM field emission gun (FEG 450, Netherlands) at CSIR-IITR, Lucknow, India. The samples were coated with gold particles using a sputter coater and analyzed in high vacuum mode and images recorded by a computer at various resolutions.

#### Cytopathological Study by Transmission Electron Microscope (TEM)

The ultrastructural changes in the cell components of virus infected poppy plants were studied using transmission electron microscope (TEM). Briefly, leaf samples of healthy and diseased samples were washed with 1X PBS (pH 7.2) prior to fixing in 2.5% glutaraldehyde prepared in sodium cacodylate buffer (pH7.2) for 2 h at 4°C. Samples were washed three times with 0.1 M sodium cacodylate buffer and post fixed in 1% osmium tetroxide for 2 h. Samples were washed with sodium cacodylate, dehydrated in acetone series (15–100%) and embedded in Araldite-DDSA mixture (Ladd Research Industries, United States). After baking at 60°C, blocks were cut (60–80 nm thick) by an ultramicrotome, (Leica EM UC7), and sections of leaf of healthy and infected plant samples were stained with uranyl acetate and lead citrate, and analyzed under G2 spirit twin TEM equipped with a Gatan digital CCD camera (FEI Tecnai, The Netherlands) operating at 60 or 80 KV. The region selected for microscopy was near the phloem tissue where the possibility of virus accumulation was highest.

### Detection of ROS

To study the cells with altered membrane permeability, samples were stained with 0.1% trypan blue in 70% ethanol followed by washings with distilled water to remove the excess stain. The blue stained cells are those with damaged membranes that allow the stain to enter the cell. Photographs were taken with an Olympus CX1 microscope fitted with Olympus digital camera (Leica Microsystems, GmbH, Germany). To detect ROS in infected and healthy cells the NBT staining was performed as described by Bournonville and Díaz-Ricci (2011).

### Nucleus Isolation and Comet Assay

For detection of apoptosis and oxidative DNA damage at the cellular level, comet assay was performed. The protoplast was isolated (Sheen, 1995) and suspended in nuclei isolation buffer (NIB) containing 0.1 mM spermidine, 10 mM MES-KOH pH 5.5, 2.5 mM EDTA, 10 mM NaCl, 10 mM KCl, 0.2 M sucrose, 0.15% Triton X-100, and 2.5 mM DTT. The solution was passed 10 times through a syringe having a 25G5/8 gauge needle. The lysate was filtered through a 20 μm Spectra Nylon Mesh filter (Spectrum Laboratories, Rancho Dominguez, CA, United States) and centrifuged at 400 ×*g* for 10 min. The pellets were suspended in 10% glycerol and stored at -20°C. Isolated nuclei were observed under an electron microscope.

For comet assay, the preparation of agarose gel for microscopic examination was carried out in three steps. In the first step, the high melting point agarose was poured on two grooves of a slide which was covered with a cover slip and stored at 4°C for 10 min. Cover slip was carefully removed and 25 μl of individually isolated nuclei from healthy and infected leaves were mixed in equal volumes with low-gelling-temperature agarose at 40°C. Next, the grooves were filled with this mixture for solidification, covered by a cover slip and stored at 4°C. The slides were dipped in 300 mM NaOH for 20 min (alkaline denaturation of nucleus) and electrophoresis was conducted at 15 V for 45 min. The slides were stained with ethidium bromide for 30 min and observed under UV light by Polarization Microscope, Leica DM2500 P (Leica Microsystems, Wetzlar, Germany). Automatic scoring of comets was performed by Comet Assay IV V4.3 (Perceptive Instruments Ltd., Suffolk, United Kingdom).

### TUNEL Assay

The infected and healthy poppy leaf samples were cut into small pieces, washed twice with PBS, and immediately fixed in paraformaldehyde (4%; w/v), and then kept in 100 mM phosphate buffer (pH 7.2) overnight. After dehydration of the samples in graded series of ethanol, they were embedded in paraffin. The sections were cut at 8 μm thickness and mounted on slides. Sections were rehydrated and incubated with proteinase-K for 15 min at 37°C. The TUNEL reaction solution (Invitrogen, United States) was mixed with the dried sample. After incubation at 37°C for 1 h, sections were rinsed three times with PBS and examined at 560 nm as a greenish fluorescence, and photographed at 40× magnification with an EVOS Cell Imaging System (Invitrogen, United States).

### Hoechst 33342 Fluorescent Staining

For detection of apoptosis at nuclear stage, dewaxing and rehydrated sections were incubated with Hoechst 33342 fluorescent dye (Thermo Scientific Pierce, United States) (1 mg ml^-1^ in PBS) for 8 min. PCD was determined by the presence of highly condensed or fragmented nuclei with blue fluorescence (340 nm excitation and 510 nm barrier filter) and photographed using EVOS Cell Imaging System.

### Analysis of Primary and Secondary Metabolites by GC-MS

Leaf and capsule samples collected at 60 and 90 days post-germination, respectively, were lyophilized for HPLC and GC-MS studies. GC-MS analysis was carried out for aqueous soluble content of infected and healthy poppy leaves and the green capsules (Bhatia et al., 2013) using Thermo Trace GC Ultra coupled with Thermo fisher DSQ II mass spectrometers. Chromatographic separations of metabolites were carried out on 30 m × 0.25 mm Thermo TR50 column (polysiloxane column coated with 50% methyl and 50% phenyl groups). X-calibur software was used to process the chromatographic and mass spectrometric data.

### Analysis of Alkaloids by HPLC

The comparative estimation of five major alkaloids namely morphine, codeine, thebaine, narcotine, and papaverine from the green capsules along with 5 cm peduncle of three biological replicates of infected and healthy poppy plants was performed (Khanna and Shukla, 1986) by HPLC (Waters, Milford, MA, United States). The calibration curves, based on absorbance, were prepared by using standard alkaloids. The alkaloid contents in the sample were calibrated with reference to the standard curve.

### qRT-PCR of Alkaloid Biosynthesis Genes

Quantitative expression of some candidate genes involved in alkaloid biosynthesis (Supplementary Table S1) by real time PCR in 20 μl reaction using Fast SYBR Green PCR Master Mix (Agilent Technologies, United States) was performed. Actin was used as an internal control to normalize signal intensity of each transcript in all the reactions. After obtaining the mean Ct value for target and endogenous reference from three biological replicates, the relative expression was calculated by 2^-ΔCt^ method.

## Results

### Disease Symptoms and Detection of Begomovirus in Poppy Samples

**FIGURE 1:**
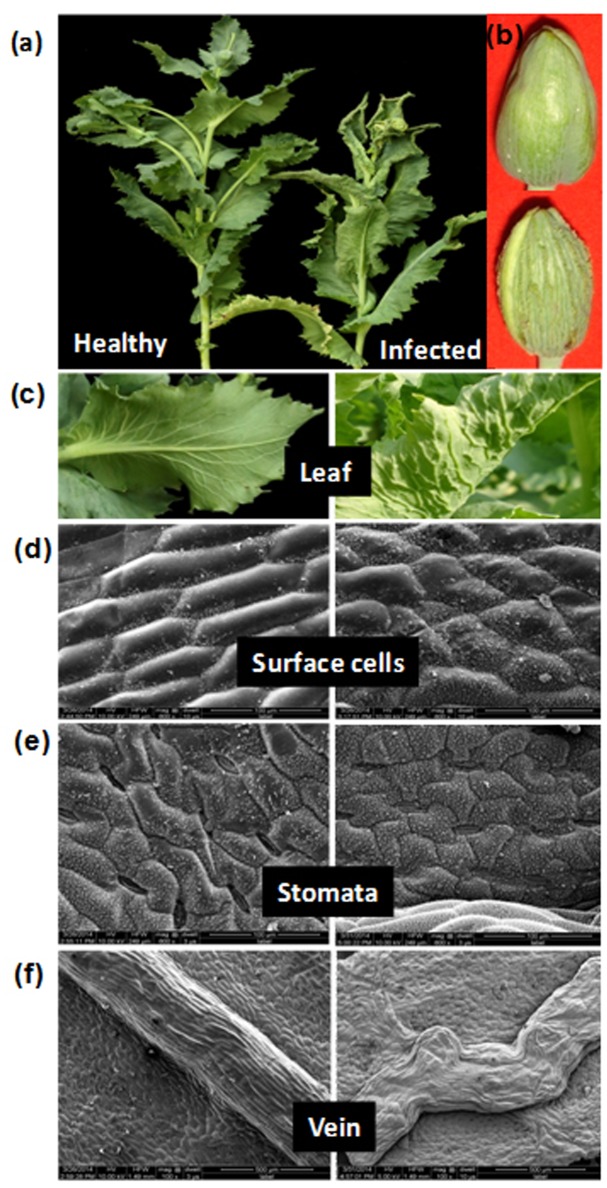
Symptoms and anatomical alterations in naturally infected *Papaver somniferum* plant. **(a)** Infected poppy plant exhibiting typical upward leaf curling, rolling, and stunting symptoms. **(b)** Capsule from same infected plant exhibiting enations. A healthy plant and capsule are shown for comparison. **(c)** Close view of healthy with no symptom and infected leaf showing upward leaf curling with enation symptoms. **(d–f)** Micrographs showing alterations in the surface cell, stomata and veins in infected leaves (right panels). **(d)** SEM of abaxial side of the leaf showing alterations in the surface cells. **(e)** Stomata are sunken. **(f)** Veins are irregularly thickened. The images on the left panels are shown as control (healthy leaves).

Symptoms of severe leaf curl and vein thickening were observed in a number of opium poppy plants. Infected plants were comparatively stunted and the leaves appeared to be narrow and small due to upward leaf curling. Leaves also exhibited thickened distorted veins on abaxial side (**Figure 1a**). The infected plants had deformed flowering buds and the capsules developed enation symptom as compared to the healthy ones (**Figure 1b**). The typical leaf curl symptoms in poppy plants suggest that the infection has been caused by begomovirus, which was confirmed by PCR analysis using begomovirus degenerate primers (Supplementary Table S1). PCR resulted in an expected size of 1.0 kb amplicon (spanning the most conserved region of virus coat protein) from the genomic DNA of 10 symptomatic (a representative shown in Supplementary Figure S1a) independent field samples (Supplementary Figure S1b). Moreover, PCR with betasatellite degenerate primers also resulted in an expected size of 1.2 kb amplicon from these 10 DNA samples (Supplementary Figure S1b). However, the presence of alphasatellite and potyvirus in these 10 samples could not be detected using their degenerate primers (Supplementary Table S1). As no mosaic symptom was observed in any of the poppy plant samples, PCR data supported the absence of potyvirus in these samples. Except for the stem where the difference is not statistically significant, the other agronomic characters like size of leaf, capsule diameter, total fresh and dry weight of plant, and total chlorophyll content of leaf were significantly reduced due to begomovirus infection as compared to that of healthy plants (Supplementary Figure S2).

### Identification of Begomovirus and Betasatellite

The full-length viral genome was amplified from a representative sample ‘PAS-1’ by RCA method which yielded the expected size of 2.7 kb DNA fragment from the amplified concatemer after restriction digestion with *Bam*HI. The 2.7 kb DNA fragment cloned in pCAMBIA1300 vector was sequenced and deposited under the accession number JQ911765. The determined 2748 nucleotides sequence revealed the presence of six potential ORFs: the V2 (pre-coat protein) and V1 (coat protein, *CP*) in virion sense strand, whereas C3 (replication enhancer protein, *REn*), C2 (transcription activator protein, *TrAP*), C1 (replication associated protein, *Rep*), and C4 (suppressor protein, *C4*) in complementary sense strand separated by an intergenic region (IR) (Supplementary Figure S3). The nonanucleotide sequence motif ‘TAATATTAC’ essentially required to bind *Rep* was present in IR which is common to all begomoviruses. The alignment of PAS-1 nucleotide sequence at BLASTn interface showed 91–99% identities with publicly available AEV sequences reported from India and Pakistan. The phylogenetic analysis corroborated well with alignment analysis and reveals the close relationship of PAS-1 sequence with AEV only (Supplementary Figure S1c). Based on high sequence identity and close phylogenetic relationships with AEV isolates, the begomovirus is identified as an isolate of AEV for which we propose the isolate descriptor AEV-[In:Lko:PS:12].

The gel purified ∼1.2 kb DNA fragment was also cloned (designated as PAS-β) and sequenced (accession number KJ948106). The full-length of cloned PAS-β was determined to be of 1362 nucleotides which harbor betaC1 ORF transcribed by complementary sense DNA strand and translated into *β-C1* protein. The alignment analysis of PAS-β nucleotide sequence revealed 93–99% identities with various Ageratum leaf curl betasatellite (ALCB) sequences. During phylogeny, the PAS-β clustered in single clad with the ALCBs reported from India and Pakistan (Supplementary Figure S1d). Based on high sequence identity and phylogeny, the PAS-β is identified as an isolate of ALCB for which we propose the isolate descriptor ALCB-[In:Lko:PS:12].

### Infectivity Test

To test the hypothesis that AEV and ALCB are the cause of severe leaf curl and vein thickening disease of poppy, infectivity tests using PAS-AEV and PAS-ALCB agroinfectious clones were performed (Supplementary Table S2). The poppy plants co-infiltrated with PAS-AEV and PAS-ALCB could develop severe leaf curl and vein thickening symptoms (Supplementary Figure S4) at 40 dpi as observed in field infected poppy plants (**Figure 1**). However, no symptoms were observed when plants were independently inoculated either with PAS-AEV or PAS-ALCB at 40 dpi. This suggests the need of AEV and ALCB for assessing symptom severity. The infectivity tests on *N. glutinosa*, *S. lycopersicon*, and on *A. conyzoides* (the original host of AEV) confirmed the infectivity of AEV (Supplementary Table S2) and pronounced the need of AEV and ALCB for severe symptom development.

### Morphological Assessment of Diseased Leaves

The visual examination of infected poppy leaves reveals upward curl and distinct vein thickening on abaxial side as compared to healthy leaves (**Figure 1c**). Morphological changes induced by AEV and ALCB infection on the adaxial surface of poppy leaves, as observed under SEM, showed disfigured and smaller cells with hyperplasia and unorganized cell arrangements (**Figure 1d**), but in healthy leaves the cells were hexagonal in shape and well arranged. The average size of cells in healthy leaves was about 125–130 μm which was reduced to 77–110 μm due to AEV infection (data not shown). The stomata on the abaxial leaf surface of infected plants were sunken and closed (**Figure 1e**) contrary to that in healthy leaves, possibly because of the severe curl, although the length of stomata was almost the same in both infected as well as healthy leaves (27–31 μm, data not shown). Moreover, veins in infected leaves also revealed hyperplasia along with wavy pattern and non-uniform thickening (from 400 to 500 μm some places) (**Figure 1f**), however, they were smooth, straight, and uniformly thickened (about of 420 μm) in healthy leaves.

### Anatomical Investigations Suggest Tissue Deformation in Leaves and Stem

Anatomy of both the stem and leaf tissues revealed structural anomalies along with the presence of dead cells in AEV infected poppy plants (Supplementary Figure S5). A well organized arrangement of cells and tissues of healthy stem (Supplementary Figure S5a) was disfigured to a great extent in the stem of infected plants (Supplementary Figure S5e). Some extracellular ducts above and below the xylem vessels along with thickened laticifers were observed adjacent or proximal to the sieve elements of phloem in the infected stem (Supplementary Figure S5e). Moreover, the epidermis of the stem was partially degenerated, disfigured and separated at many places from the hypodermis in AEV infected plants (Supplementary Figure S5f) while, was well in architecture as a distinct single layer in stem of healthy plants (Supplementary Figure S5b). The vascular bundles were disorganized, xylem cells were highly lignified and phloem cells had collapsed and were indistinguishable from the laticifer cells (Supplementary Figure S5g) compared to that of the healthy stem (Supplementary Figure S5c). The pith cells of infected stem were flaccid, had lost their membrane integrity and were difficult to recognize (Supplementary Figure S5h) than stem of healthy plants (Supplementary Figure S5d). The hypertrophied cortical parenchyma in infected stem had fewer intercellular spaces and deformed cells with thickened walls (data not shown).

Likewise, the anatomical study of the midrib of AEV infected poppy leaves showed deep stained (with trypan blue) vascular bundles and surrounding cells (Supplementary Figures S5k,l) as compared to healthy ones (Supplementary Figures S5i,j). Excessive staining in infected tissues indicates the damaged plasma membranes, and also reveals loss of membrane integrity, eventually causing them to lose their shape and retain the blue stain, suggesting induced cell death in AEV infected plants (Supplementary Figures S5k,l). The unevenly arranged and poorly developed vascular bundles of leaf midrib and veins lead to the formation of enation and curl symptoms.

### TEM Analysis Suggests PCD, Validated by Comet Assay

**FIGURE 2:**
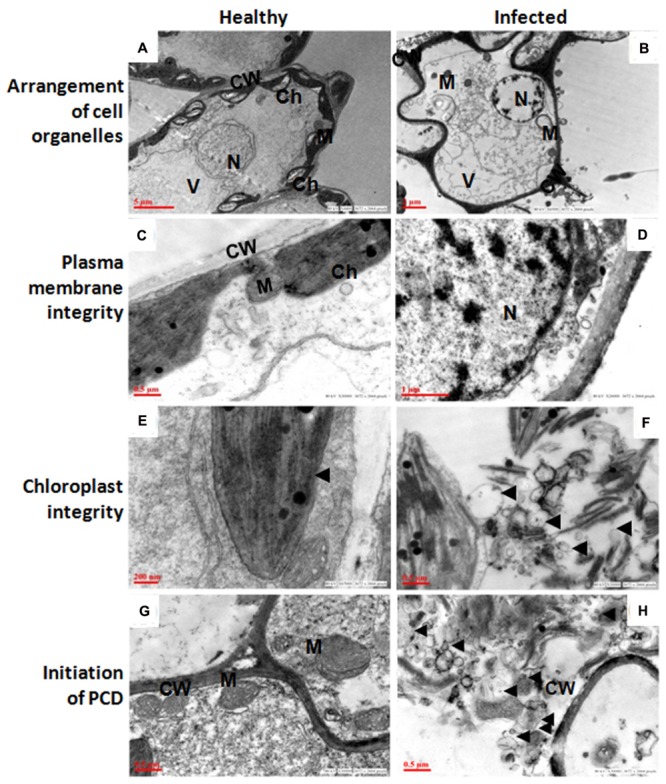
*Ageratum enation virus* (AEV) infection disturbed arrangement of cell organelles, integrity of plasma membrane and chloroplast, and accelerate cell death. **(A,C,E,G)** TEM images of thin sections of healthy poppy leaf showing structural integrity and status of normal cells. **(B)** TEM images of AEV infected poppy leaf showing the disturbed compartmentalization and integrity of the cell resulting in pleomorphic cell shape. **(D)** Cell membrane detachment from the cell wall. **(F)** Punctured chloroplasts in infected cells. **(H)** Infected cells undergo degradation of cell organelles and cell wall. Black arrows show detachment of cell membrane from wall and bleb’s formation. N, nucleus; M, mitochondria; Ch, chloroplast; V, vacuoles; CW, cell wall.

The ultra-structural analysis of AEV infected and healthy leaf of poppy plants was performed using TEM, which revealed significant changes in the cell organelles (**Figure 2**). Disturbance in the integrity of cell and uneven thickness of cell wall was observed in the AEV infected sample, (**Figure 2B**) compared to that of healthy ones (**Figure 2A**). In the infected leaf tissue, the plasma membrane was partially or completely detached from the cell wall. However, in the healthy tissue the plasma membrane was present as a thin lining along with the inner cell wall. The disintegration and detachment of plasma membrane, loss of the structural integrity of mitochondria (**Figure 2D**), fragmentation of intracellular organelles such as the nucleus and chloroplast (**Figure 2F**), and production of blebs and membranous vesicles by collapsing cells, (**Figure 2H**) clearly indicated induced PCD (apoptosis) in AEV infected cells, and were comparable with the healthy cells (**Figures 2C,E,G**). In AEV infected tissue, severely damaged thylakoids and grana were observed which extruded into the cytoplasm from ruptured chloroplast wall (**Figure 2F**), however, chloroplast in healthy leaf tissue was membrane bound, intact and distinct (**Figure 2E**). Abnormal building up of oily inclusions and empty vacuoles in the chloroplast stroma were also observed. Along with the drastically altered chloroplasts, disrupted outer membrane and cristae of the mitochondria showed that infected cells underwent final stages of apoptosis. The TEM study showed that dying cells contain small discrete apoptotic bodies formed due to the disintegration of organelles (**Figure 2H**). Our studies suggested that infection by AEV causes induced apoptosis, however, the cells of healthy plants of the same age revealed distinct cellular organization.

**FIGURE 3:**
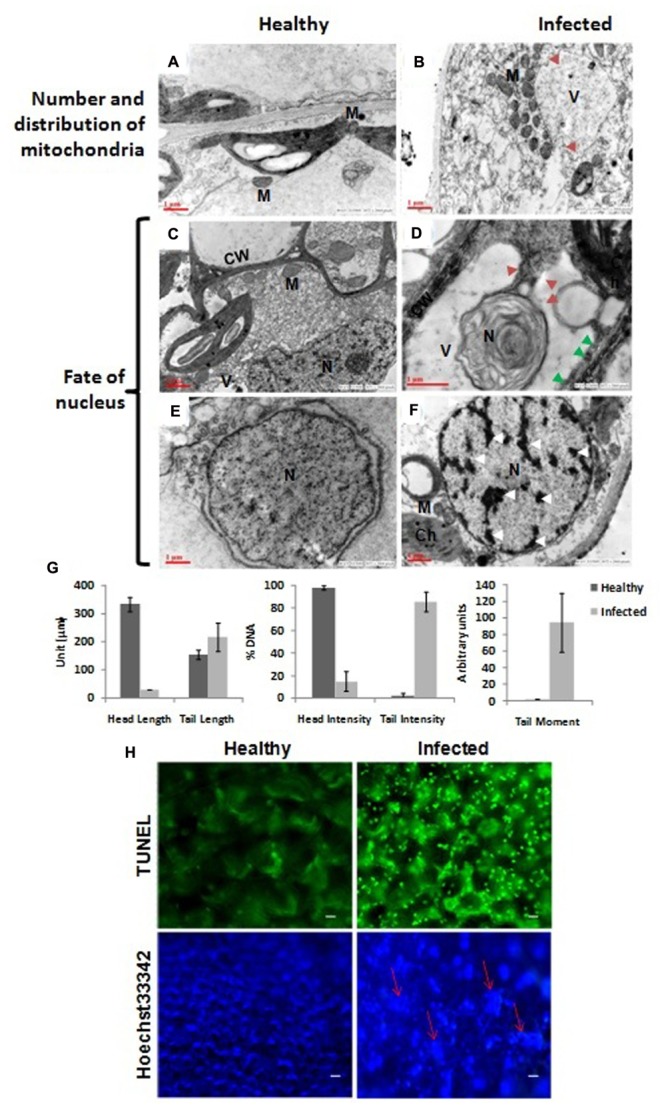
Virus infection induced programmed cell death (PCD) in poppy plants. **(A,B)** In control cells the mitochondria are distributed throughout the cells while they surround the vacuole in infected cells and the vacuole is forming the autophagosomes by initiating invagination (red arrows). **(D)** The vacuole engulfing a nucleus in an autophagic cell also showing apoptotic bodies (solid red arrow) and degraded cell wall (solid green arrows) as compared to healthy **(C)**. **(E,F)** Chromatin condensation and movement toward the periphery (shown by white arrows). N, nucleus; M, mitochondria; Ch, chloroplast; V, vacuoles; CW, cell wall. **(G)** Graphical presentation of single cell electrophoresis of isolated nucleus from healthy and infected leaf tissue, analyzed by Comet Assay IV^TM^ V4.3 software. Each data are expressed as means ± SE of at least 3 nt biological replicates. **(H)** Detection of PCD using TUNEL assay. Upper panel: Strong green fluorescence (at 560 nm) correlating well with more DNA cleavage, due to induced PCD, in AEV infected leaf tissue. Lower panel: Confirmation of PCD by Hoechst 33342 dye staining assay showing more blue fluorescence (at 340 nm) due to highly condensed or fragmented nuclei (shown by red arrows) of AEV infected leaf. Bar = 5 μm.

The vacuoles in infected cells were very large and displayed phagosomic structures which formed near the periphery, and were surrounded by a large number of mitochondria (**Figure 3B**) while no such features were observed in healthy cells (**Figure 3A**). **Figure 3D** clearly shows a large invaginating vacuole engulfing the nucleus of an infected cell confirming the PCD in AEV infected cells, however no such alterations were observed in cells of healthy tissue (**Figure 3C**). The reduction in cell volume (cell shrinkage), nuclear pyknosis or chromatin condensation and their distribution at the periphery was also apparent in AEV infected cells (**Figure 3F**), while these were absent in control tissue (**Figure 3E**). Furthermore, the comet assay suggested that AEV infection increased double-strand breaks in poppy (**Figure 3G**) possibly due to accelerated PCD at a single cell level in virus infected tissues. Comet assay resulted in low head length, high tail moment, and strong tail intensity in the AEV infected nucleus, suggesting a high degree of degradation of nuclear DNA in AEV infected tissue (**Figure 3G**). The results clearly establish that AEV infection cause DNA breaks in infected tissues.

To further confirm the PCD, TUNEL assay was applied which can efficiently detect the cells that undergo extensive DNA degradation during the late stages of apoptosis by labeling the 3′-OH termini in the double-strand DNA breaks generated (Kyrylkova et al., 2012). To detect the apoptotic cell death in AEV infected plants, leaf sections were treated with TUNEL reagent and counterstained with Hoechst 33342. TUNEL reagent only stains the apoptotic cells, while the Hoechst 33342 stains the DNA of all cells. Therefore, a strong fluorescence was clearly detected in the AEV infected leaf tissue (**Figure 3H**, upper panel), correlating the DNA cleavage with induced PCD during this stage of detection. On the other hand, control (healthy leaves) when stained with TUNEL reagent did not produce fluorescence. As controls, nuclei from both infected and uninfected healthy leaves were stained using Hoechst 33342 dye, as shown in (**Figure 3H**, lower panel). The nuclei of cells in AEV infected leaves showed a blue fluorescence whose intensity was more than that in the uninfected leaves.

### Elevated Production of Reactive Oxygen Species (ROS) in AEV Infected Tissue

Reactive oxygen species (ROS) are generated as toxic by-products of aerobic metabolism. They play an important role in signaling network and regulating numerous biological processes such as growth, development, response stresses, and PCD (Bailey-Serres and Mittler, 2006; Del Río, 2015). These harmful species are removed by means of antioxidants and antioxidative enzymes. Herein, the antioxidant enzyme assays were performed biochemically and by NBT staining method to see the effect of AEV infection in poppy plants. The production of MDA and proline increased by 1.25- and 4.53-fold in infected tissue as compared to that in healthy plants (Supplementary Figure S6). Moreover, the activities of ubiquitous antioxidant enzymes: CAT, SOD, and APX were also enhanced by about 1.5-fold, which possibly may help in ROS scavenging (Supplementary Figure S6). NBT staining assay was performed for assessing the production of ROS, and this corroborated with the earlier findings which showed that excess ROS production leads to loss of structural integrity in the phloem of infected tissue. Further, using light microscopy necrosis was also observed in infected leaf tissue, which is a strong indicator of induced PCD (data not shown).

### AEV Infection Alters Alkaloid Contents and Expression of Their Biosynthetic Pathway Related Genes

**FIGURE 4:**
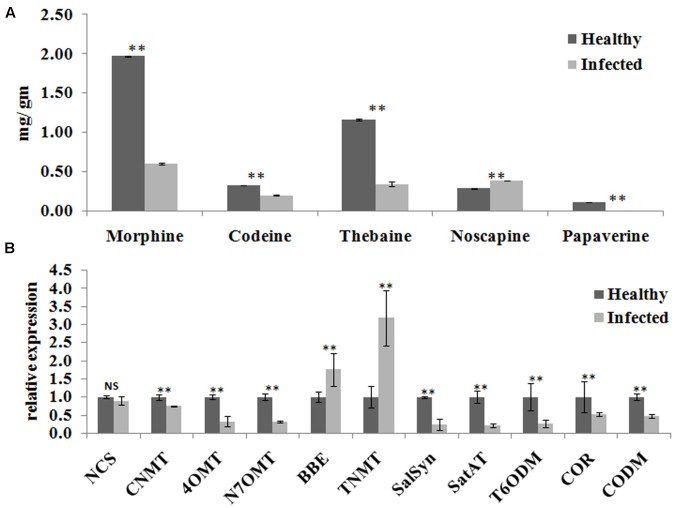
*Ageratum enation virus* infection altered the alkaloid contents and perturbs the respected pathway genes in infected poppy sample. **(A)** HPLC analysis revealed the alterations in major alkaloids, morphine, codeine, thebaine, narcotine, and papaverine in infected plant. **(B)** qRT-PCR analysis confirmed the HPLC data and revealed perturbation in expression of structural genes involved in the biosynthetic pathway of alkaloids of infected poppy plants. Values are given as the mean ± StDev of three independent experiments. Asterisks indicate statistically significant differences by the *t*-test at *p* ≤ 0.01. NCS, norcoclaurine synthase; CNMT, coclaurine *N*-methyltransferase; 4′OMT, 3′-hydroxy-*N*-methyl coclaurine 4′-*O*-methyltransferase; N7OMT, norreticuline 7-*O*-methyltransferase; BBE, berberine bridge enzyme; TNMT, tetrahydroprotoberberine *cis-N*-methyltransferase; SalSyn, salutaridine synthase; SalAT, salutaridinol 7-*O*-acetyltransferase; T6ODM, thebaine 6-*O*-demethylase; COR, codeinone reductase; CODM, codeine *O*-demethylase.

Yield of medicinal alkaloids is a major concern in opium poppy which decides their economic value. Therefore, HPLC analysis was performed to quantify the changes in alkaloid levels in AEV infected plants. The alkaloid profiling of capsules clearly demonstrates 69, 38, and 71% decrease in the levels of morphine, codeine, and thebaine, respectively, in infected samples as compared to that in the healthy ones (**Figure 4A**). The biosynthesis of papaverine alkaloid in the AEV infected capsules was completely stopped and an increase in noscapine content was observed (**Figure 4A**).

**FIGURE 5:**
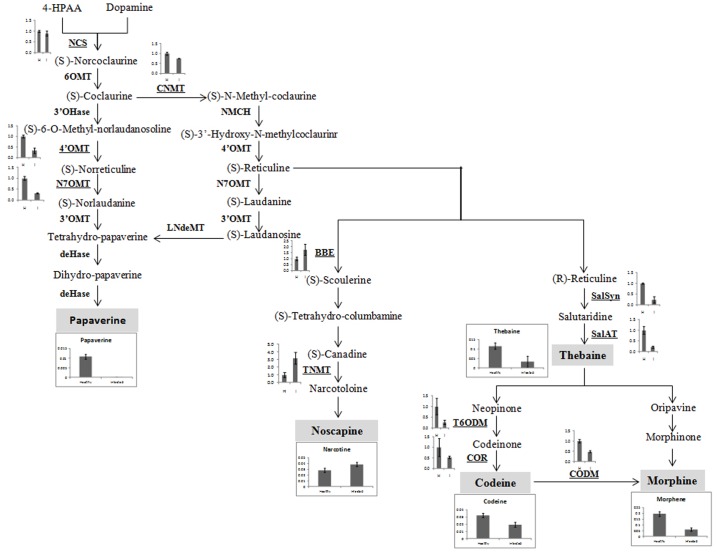
Biosynthetic pathway of papaverine, noscapine, thebaine, codeine, and morphene alkaloids showing perturbation in expression of genes involved in their biosynthesis in AEV infected opium poppy plant. The major steps in alkaloid biosynthesis are shown here. The genes assessed by qRT-PCR have been bold underlined and obtained results have been shown near to them. The major alkaloids (shown in gray shadowing) and qRT-PCR of their respective genes has also been shown here. NCS, norcoclaurine synthase; CNMT, coclaurine *N*-methyltransferase, 4′OMT, 3′-hydroxy-*N*-methyl coclaurine 4′-*O*-methyltransferase; N7OMT, norreticuline 7-*O*-methyltransferase; TNMT, tetrahydroprotoberberine *cis-N*-methyltransferase; BBE, berberine bridge enzyme; SalSyn, salutaridine synthase; SalAT, salutaridinol 7-*O*-acetyltransferase; T6ODM, thebaine 6-*O*-demethylase; COR, codeinone reductase; CODM, codeine *O*-demethylase.

The expression of major genes involved in the biosynthesis of major alkaloids by qRT-PCR supported the results of HPLC analysis. The genes norcoclaurine synthase (NCS), acetyl-CoA-dependent salutaridinol-7-*O*-acetyltransferase (SalAT), thebaine 6-*O*-demethylase (T6ODM), NADPH-dependent codeinone reductase (COR), codeine-*O*-demethylase (CODM), norreticuline 7-*O*-methyltransferase (N7OMT), salutaridine synthase (SalSyn), 3′-hydroxy-*N*-methyl coclaurine 4′-*O*-methyltransferase (4′OMT) involved in biosynthesis of (S)-norcoclaurine, thebaine, codeinone, codeine, papaverine, and morphine, respectively, were found to be downregulated in infected plants. The expression of berberine bridge enzyme (BBE) and tetrahydroprotoberberine *N*-methyltransferase (TNMT), the important genes involved in biosynthesis of noscapine, was found upregulated by about twofolds (**Figures 4B**, **5**). As a result, it may be assumed that due to AEV infection, the expression of noscapine and related genes (BBE and TNMT) are enhanced to protect the opium poppy plant (**Figure 5**).

### AEV Infection Alters Metabolic Activities in Infected Plants

**FIGURE 6:**
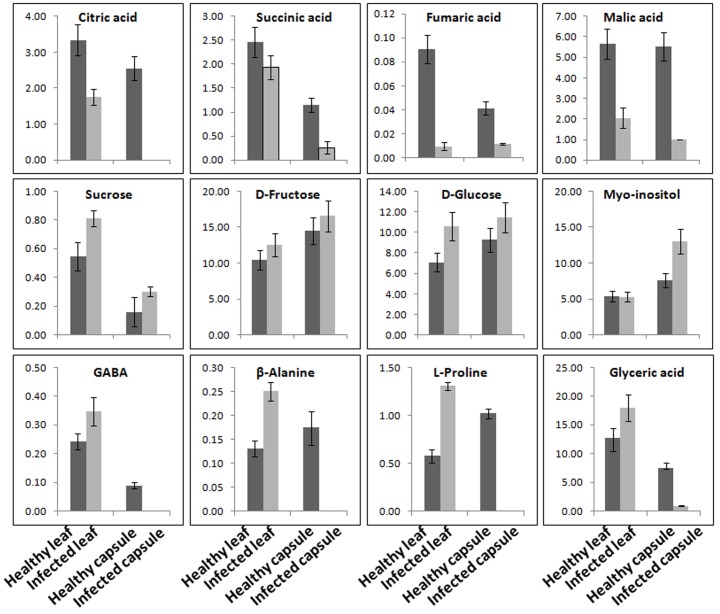
*Ageratum enation virus* infection altered the major metabolite contents involved in normal plant development and defense. GC-MS data analysis suggesting the considerable alterations in the metabolite contents of TCA cycle, sugars and stress related enzymes in leaf and capsule of infected poppy plant as compared to healthy controls. Data are given as means ± SEM, which were calculated using three biological replicates.

Aqueous extracts of leaves and capsules obtained from the AEV infected and healthy plants were analyzed by GC-MS. A total 38 chemically diverse metabolites including amino acids, sugars, organic acids, and amines were identified (**Figure 6** and Supplementary Table S3). The concentration of metabolites varied in healthy and infected plants. Most of the metabolites of TCA cycle intermediates and those of organic acids such as malic acid, L-threonic acid, citric acid, D-fructo furanose, succinic acid, L-tartaric acid, D-glucuronic acid, and 2-ketogluconic acid were either detected in low quantities or were not detectable in capsules as well as leaves of AEV infected plants. Moreover, a significant reduction in morphine content also was observed in capsules of infected plants. Aqueous extract of capsules of the AEV infected plants showed higher contents of D-fructopyranose, D-galactose, D-gluconic acid, α-D-glucopyranose, myo-inositol, galactose oxime, D-glucose, D-glucosamine and sucrose, while their levels were reduced or unchanged in infected leaves of the same plant. Increase in phosphoric acid, D-fructose, D-fructosemethyloxime (Syn), and D-glucose was more or less the same in both capsules and leaves of virus infected plants. On the other hand, only four metabolites: glyceric acid, GABA, β-alanine and L-proline showed increased concentration in the infected leaves, while they were not detected in infected capsules. These findings suggest virus induced alkaloid and metabolite perturbations in infected tissues.

## Discussion

*Ageratum enation virus* and associated satellite DNAs (ALCB and ALCA) were originally discovered from a weed plant, *A. conyzoides* (Tahir et al., 2015). Now, AEV is emerging as a devastating pathogen in India and reported to cause significant losses in many crops (Kumar et al., 2013; Srivastava et al., 2013; Swarnalatha et al., 2013). The present study identified the association of AEV and ALCB with severe leaf curl and vein thickening disease of opium poppy for the first time. Prior to this, it was reported as a host of a bipartite DNA begomovirus (Srivastava et al., 2016) and RNA viruses (Pethybridge et al., 2005; Zaim et al., 2014). The applicability of agroinfiltration assay in opium poppy plants has been shown as our results of infectivity assay using the agroinfectious cloned DNAs of AEV and ALCB could induce the symptoms as were observed in naturally infected poppy plants. Our study revealed the severe anatomical changes like deformed cells in epidermis layer, cortical parenchyma and connective tissues with thick walls of stem and leaf tissues, besides the morphological alterations in infected plants. Our study is well supported by the earlier findings where *Jatropha* and *Aamaranthus* plants infected by *Jatropha mosaic virus* and AEV, respectively, have been shown structural alterations and disruption of vascular bundles in stem and leaf tissues using HR-MAS NMR spectroscopy and MR imaging methods (Sidhu et al., 2010; Srivastava et al., 2012). The infection of *Beet severe curly top virus* has shown to induce the hyperplasia in inoculated *Arabidopsis* (Lai et al., 2009) strengthened our finding of induced hyperplasia in opium poppy where undifferentiated small cells with non-uniform arrangements in leaf and stem tissues was observed. Laticifers rest adjacent to the sieve elements of the phloem in the opium poppy and are the main channels where alkaloids accumulate in the form of latex (the cytoplasm of highly specialized laticifer cells) (Onoyovwe et al., 2013). It was observed that AEV infection induced the formation of laticifers due to the accelerated autophagic activities and evidenced by the dark staining of the phloem region. Earlier, Marty (1999) also observed that stressful environment trigger the autophagic activity in poppy plants and revealed the degradation of cytoplasm in the central region of the cells which led to the formation of more laticifers.

Overabundance of ROS can initiate a variety of auto-oxidative chain reactions of membrane unsaturated fatty acids and generate lipid hydroperoxides. The cascades of events led to the destruction of organelles and macromolecules (Moshe et al., 2012). The TEM analysis of AEV infected poppy leaf clearly demonstrated the damage in mitochondria and chloroplast due to the higher accumulation of ROS and provide the evidence of accelerated PCD in cells to avoid further spread of the AEV. PCD is also reported to initiate from the virus infected phloem or cambium cells and spreads to other nearby infected cells (Miozzi et al., 2014). A higher number of deep blue stained cells, detected by the NBT staining assay, near the phloem also confirm the AEV induced ROS production in poppy. Under stress, higher proline accumulation is a common physiological response to scavenge the elevated ROS in plant system to prevent the induction of PCD (Moshe et al., 2012). In the AEV infected poppy sample, the production of stress biomolecules like sugar and proline was elevated by twofold. Moreover, the production of scavenging enzymes APX, LPX, SOD, and CAT was also elevated to cope with the elevated ROS. The role of these scavenging enzymes in reduction of virus induced ROS has been shown earlier (Kumar et al., 2016) and supported our analysis.

The PCD causes chromatin condensation, chromosomal DNA fragmentation, and internucleosomal fragmentation of nuclear DNA (Asada, 2006; Miozzi et al., 2014), where DNA is the important target for double- and single-strand breaks (Gyori et al., 2014). To detect the fragmentation of nuclear DNA, the use of the comet and TUNEL assays have widely increased in plant system as only a small number of cells are required to obtain the reliable results (Charzyńska et al., 2000; Langie et al., 2015). The comet assay is a rapid, visual, and sensitive technique used to measure DNA damage (DNA breakage or formation of alkali-labile sites) in individual cells (McKelvey-Martin et al., 1993). Electrophoresis of supercoiled DNA nucleoid at high pH results in structures resembling comets, observed by fluorescence microscopy, where the intensity of the comet tail relative to the head reflects the number of DNA breaks because the loops containing a break lose their supercoiling ability and become free to extend toward the anode in form of tail. Results of the comet assay in our study revealed a significant low head length in the AEV infected nucleus while the tail moment was not as high as expected, but the tail intensity was strong enough to suggest degradation and fragmentation of DNA. The tail intensity (percentage DNA in the tail) occurs due to conversion of alkali-labile sites to strand breaks and is considered to be the most informative measurement for confirming PCD (Collins et al., 2014). While tail length is not statistically different between healthy and infected plants, the tail moment and other measurements clearly support PCD hypothesis. Moreover, the tail length measurement has limitation as the length reaches a plateau at quite low levels of damage (Collins, 2004) and is also sensitive to background intensity of an image analysis system which affects the criteria for determining the end of the tail (Collins et al., 2014). Following to this, AEV induced accelerated PCD was also confirmed by the TUNEL assay (Kyrylkova et al., 2012) where DNA fragmentation, chromosomal condensation, and shrinkage of nuclei were observed. TUNEL dye only stains the apoptotic cells by labeling the 3′-OH termini in the double-strand DNA breaks generated and therefore strong signals could be observed in AEV infected leaf tissue only. While, Hoechst 33342 stains the DNA of all cells, but could clearly demonstrate the chromosomal condensation in infected cells. In a study, Yao et al. (2002) also demonstrated the DNA cleavage in the nuclei of the Rye mottle virus infected cells by observing the distribution of EM-TUNEL-positive gold particles in the condensed chromatin and suggested apoptotic cell death in the yellow portion of the virus infected oat leaves. These findings corroborate well with our findings and support the AEV induced accelerated PCD in the infected poppy plant.

Both HPLC and GC-MS approaches have been shown to determine the relative level of metabolites across the viral infection processes (Kogovšek et al., 2016). HPLC analysis of alkaloids revealed that the AEV infection tends to downregulate the biosynthesis of morphine, thebaine, codeine, and papaverine alkaloids, except the Noscapine whose biosynthesis was marginally upregulated. The qPCR of some pathway genes like NCS, CNMT, SalSyn, SatAT, T6ODM, COR, CODM showed downregulation and this corroborated with the findings of HPLC analysis where quantities of these gene products was found to be low. Winzer et al. (2012) compared three traits of poppy in HM1, HT1, and HN1 lines synthesizing morphine, thebaine, and noscapine, respectively, in large quantities, and showed that when noscapine is high in capsule, the morphine is low, presumably due to the substrate competition. They also silenced the morphine pathway by virus-induced gene silencing method and showed the accumulation of noscapine pathway intermediates. The substrate (S)-Reticulin is reported as a common substrate in the pathway of alkaloid biosynthesis (Winzer et al., 2012). These findings support our data that higher noscapine biosynthesis may be due to the higher availability of (S)-Reticulin in AEV infected tissue. Further, the expression of noscapine intermediate biosynthesis genes (like BBE and TNMT) was found to be enhanced in AEV infected tissue, and this correlates with the high noscapine production. An earlier study revealed the increasing trend for morphine, codeine, and papaverine alkaloids due to PMV-P infection (Zaim et al., 2014) possibly due to severe structural deformity in phloem cells of leaf and stem tissue of AEV infected opium poppy, which contradict our findings (Supplementary Figure S5). The GC-MS data of AEV infected tissue showed reduction in most of the intermediate compounds of the TCA cycle and organic acids. The study on *Potato virus Y* infected potato plants showed an initial decrease in the concentrations of metabolites connected with sugar and amino-acid metabolism and TCA cycle (Kogovšek et al., 2016) and corroborates the present findings. The accumulation of TCA cycle intermediates, such as citrate and malate was significantly increased in JMV-infected *J. cursus* plants (Sidhu et al., 2010). Higher accumulation of an important cellular metabolite, myo-inositol was observed which suggests the activation of the plant defense system in AEV infected poppy plants (Eckardt, 2006). Both the GABA and β-alanine are non-protein amino acids which generally accumulate in response to biotic and abiotic stresses (Zulak et al., 2008; Kogovšek et al., 2016). Higher levels of GABA and β-alanine amino acids were reported in infected leaves of poppy, while they were not detected in infected capsules. The probable cause for this may be the inhibition of translocation of metabolites from leaves to capsule, due to phloem breakage and presence of intensified laticiferous tissue.

Plants are able to modulate their sugar pools as a fuel for metabolism or as a signal to intensify immune reactions (Moghaddam and Van den Ende, 2012). The GC-MS study of AEV infected poppy leaves and stem tissues demonstrated 1.5-fold upregulation in sugar derivatives, which suggested that infected plants divert their carbohydrate flux from metabolic activities to sensing the stress (Gómez-Ariza et al., 2007). The other possibility of increased sugar is gluconeogenesis, reprogramming the synthesis of glucose from non-carbohydrate sources as was observed in TGMV and *Tomato yellow leaf curl virus* (Moshe et al., 2012) infected tomato leaves. Additionally, leaf samples of AEV infected plants showed significant reduction in organic acid contents such as pyruvate, lactate, glycerol and they may be converted into carbohydrate by gluconeogenesis. PCA of metabolites of AEV infected leaves and capsules when compared with their healthy controls showed high metabolite perturbations in AEV infected capsules, while inin controls there were no such signs (Supplementary Figures S7, S8). This study clearly demonstrated that AEV infection causes reduction in the metabolite contents as shown by GCMS and HPLC analysis, resulting in decrease in the commercial value of the crop. To the best of our knowledge this is the first comprehensive study of immune response of opium poppy plant against the begomovirus infection.

## Author Contributions

SKR conceived the idea for work. AS, LA, RR, SG, MJ, RD, and TT have performed the experiments. AS, LA, SK, and SKR have written the MS. PCS has done microscopy. BS performed TUNEL and Hoechst 33342 fluorescent staining assays. PSC has done statistical analysis of the data. TT and OS have contributed in GC-MS analysis. SS provided opium poppy plants and did HPLC analysis.

## Conflict of Interest Statement

The authors declare that the research was conducted in the absence of any commercial or financial relationships that could be construed as a potential conflict of interest.

## Footnotes

1 http://www.geograph.org.uk/photo/4520553

2 www.ncbi.nlm.nih.gov/projects/gorf/
