# Supplementary material for: Ageratum enation virus Infection Induces Programmed Cell Death and Alters Metabolite Biosynthesis in Papaver somniferum
Source: Front Plant Sci. 2017 Jul 6;8:1172. doi: 10.3389/fpls.2017.01172 (PMC5498505; doi:10.3389/fpls.2017.01172)
Supplement: Supplementary file 1 [file Data_Sheet_1.DOC]

***Ageratum enation virus* infection induces programmed cell death and alters metabolite biosynthesis in *Papaver somniferum***

**Running title: AEV induced PCD in poppy**

Ashish Srivastava^1,6†^, Lalit Agrawal^2†^, Rashmi Raj^1^, Meraj Jaidi^1^, Shri Krishna Raj1, Swati Gupta^2^, Ritu Dixit^2^, Poonam C. Singh^2^, Tusha Tripathi^3^, Om Prakash Sidhu^3^, Brahma N. Singh,^4^ Sudhir Shukla,^5^ Puneet Singh Chauhan^2^, Susheel Kumar^1^*

^1^Plant Molecular Virology Laboratory, CSIR-National Botanical Research Institute, Rana Pratap Marg, Lucknow-226001, India;

^2^Division of Plant Microbe Interaction, CSIR-National Botanical Research Institute, Rana Pratap Marg, Lucknow-226001, India;

^3^Phytochemistry Division, CSIR-National Botanical Research Institute, Rana Pratap Marg, Lucknow-226001, India;

^4^Pharmacognosy and Ethnopharmacology Division, CSIR-National Botanical Research Institute, Rana Pratap Marg, Lucknow-226001, India;

^5^Plant Breeding Laboratory, CSIR-National Botanical Research Institute, Rana Pratap Marg, Lucknow-226001, India

^6^Amity institute of Virology and Immunology, Amity University, Noida-201303, India

^†^These authors contributed equally to this work.

*Corresponding author:

Susheel Kumar;

E-mail: susheelnbri@gmail.com; Tel: +91-522-2297951

**Supplemental information data (Method S1, Table S1-S3 and Figure S1-S8)**

**Method S1: Supplemental Experimental Procedures**

**Estimation of Chlorophyll pigment content**

Chlorophyll was extracted from opium poppy leaves using 80% acetone as solvent. Briefly, 0.3 g fresh sample was homogenized in 3.0 ml (v/v) of 80% chilled acetone. The absorbance was taken at 645nm and 663nm in a UV-160 spectrophotometer for Chl*a* and Chl*b* respectively, and chlorophyll contents were calculated using the equations suggested by Lichtenthaler and Buschmann (1987) as

Chl*a* (mg g^-1^ FW) = (11.75 x A_663_ - 2.35 x A_645_) x V/(1000 × w)

Chl*b* (mg g^-1^ FW) = (18.61 x A_645_ - 3.96 x A_663_) x V/(1000 × w)

Where,

A_645_ = absorbance at a wavelength of 645 nm;

A_663_ = absorbance at a wavelength of 663 nm;

V = final volume of 80% acetone (ml);

w = dry weight of sample taken (g)

**Proline content**

The proline content was quantified by acid-ninhydrin procedure described earlier (Bates *et al*., 1973). The leaf tissue (0.5 g) was ground with 3% sulfosalicylic acid (10 ml) and clarified by centrifugation. Supernatant (2.0 ml) was mixed with equal volume of acid-ninhydrin and acetic acid (v/v), the mixture was incubated at 100 °C for 1 h, and the reaction was finished in an ice bath. The reaction mixture was extracted with 4.0 ml of toluene using a vortex mixer for 15-20 sec and absorbance was read at 520nm.

**Lipid peroxidation (Malondialdehyde, MDA)**

Lipid peroxidation was determined by the estimation of MDA content following the procedure described elsewhere (Heath & Packer, 1968) with slight modification. Plant material 0.5 g was homogenized in 5 ml of 0.1% trichloroacetic acid (TCA). The homogenate was centrifuged at 10000 x g for 5 min for every 1 ml of aliquote, 4.0 ml of 20% TCA containing 0.5% thiobarbitaric acid (TBA) was added. Mixture was heated at 95 ^o^C for 30 min and then cooled quickly on ice-bath. After centrifugation at 10000 x g for 15 min the absorbance of the supernatant was taken at 532nm and 600nm.

**Antioxidant enzyme assays**

A crude enzyme extract was prepared by homogenizing 0.5 g of frozen leaf tissues in an extraction buffer containing 1 mM EDTA, 0.05% Triton X-100 and 2% polyvinyl pyrrolidone (PVP), 1.0 mM ascorbate in 50 mM potassium phosphate buffer (pH 7.8) using a chilled mortar and pestle. The homogenate was centrifuged at 10,000 g for 20 min and the supernatant was stored at -20 ^o^C for further used for the following enzyme assays.

**Superoxide dismutase (SOD) activity**

Total SOD activity was determined following the procedure described earlier (Beauchamp & Fridovich, 1971). The reaction mixture contained 13 mM methionine, 2 mM riboflavin, 0.1 mM EDTA, and 75 µM nitrobluetetrazolium (NBT) salt dissolved in 3.0 ml of 50 mM sodium phosphate buffer (pH 7.8). The 3.0 ml of the reaction mixture was added to 100 µl of enzyme extract. The mixtures were illuminated in glass test tubes in triplicates of Philips 40-W fluorescent tubes. The absorbance was read at 560 nm in the spectrophotometer against the blank. The SOD activity is expressed in U mg^–1^ protein (U = change in 0.1 absorbance h^–1^ mg^–1^ protein under assay conditions).

**Catalase (CAT) activity**

Total CAT activity was assayed according to the method of Chandlee and Scandalios (1984). The assay mixture contained 2.6 ml of 50 mMl^–1^ potassium phosphate buffer (pH 7.0), 0.4 ml of 15 mMl^–1^ H_2_O_2_, and 0.04 ml of enzyme extract. Catalyse mediated changes in absorbance were read at 240 nm. The enzyme activity was expressed in U mg^–1^ protein (U = 1 mM of H_2_O_2_ reduction min^–1^ mg^–1^ protein). The enzyme protein was estimated by the method of Bradford (Bradford, 1976) for all the enzymes.

**Ascorbate peroxidase (APX) activity**

The APX activity was determined spectrophotometrically by recording a decrease in the absorbance at 290 nm because of oxidation of ascorbate (Lichtenthaler & Buschmann, 1987). The 3.0 ml of reaction mixture contain 50 mM phosphate buffer (pH 7.0), 0.1 mM H_2_O_2_, 0.5 mM sodium ascorbate, 0.1 mM EDTA and a suitable amount of enzyme extract. One unit of APX activity was assumed as the amount of the enzyme which oxidized 1 μM ascorbate per min at 30 ^o^C.

**Agroinfectious clones for transient expression of viral DNAs**

To determine the infectivity, infectious clones of AEV and ALCB with tandem repeats were constructed in pCAMBIA1300 vector. The full-length AEV genome (cloned at *Bam*HI site of pCAMBIA1300, clone PAS-A) was sequentially digested with *Eco*RI followed by *Bam*HI enzymes to release the partial genome of 1568 nucleotides. This partial genome was gel purified, and ligated at *Eco*RI and *Bam*HI site in pCAMBIA1300 to develop PAS1-Temp clone using the standard cloning method. To the PAS1-Temp, the full-length AEV genome (of 2748 nucleotides) was subsequently ligated at *Bam*HI site to develop the tandem repeat in such a way that when it replicate it may synthesize the complete viral genome to cause the infection in test plants, and designated at PAS-AEV. For infectious clones of ALCB, the full-length ALCB genome (cloned in pGEM-T vector, clone PAS-β) was sequentially digested with *Kpn*I and *Cla*I enzymes to release the partial genome of 810 nucleotides which was ligated in pCAMBIA1300 to develop the PAS-β-Temp clone. Then, a full-length *Kpn*I digested ALCB genome was subsequently ligated at PAS-β-Temp clone to develop the PAS-ALCB clone with tandem repeats of ALCB. The method described earlier (Srivastava et al. 2013) was followed to develop the agroinfectious clones in *Agrobacterium tumefaciens* strain GV3101. The syringe infiltration method was used to infiltrate four- to six-leaf stage *P. somniferum*, *Nicotiana glutinosa*, *Solanum lycopersicon* and *Ageratum conyzoides* test plants. The infiltrated plants were kept in glasshouse conditions (natural illumination with temperature of 25±2°C) and examined regularly for appearance of symptoms.

**References**

Bates, L., Waldren, R. P., Teare, I. D. (1973). Rapid determination of free proline for water-stress studies*. Plant Soil* 39, 205-207.

Beauchamp, C., Fridovich, I. (1971). Superoxide dismutase: Improved assays and an assay applicable to acrylamide gels. *Analytical Biochemistry Review* 44, 276-287.

Bradford, M. M. (1976). A rapid and sensitive method for the quantification of microgram quantities of protein utilizing the principle of protein-dye binding. *Analytical Biochemistry* 72, 248-254.

Bull, S. E., Briddon, R. W., Markham, P. G. (2003). Universal primers for the PCR-mediated amplification of DNA 1: A satellite-like molecule associated with begomovirus-DNA β complexes. *Molecular Biotechnology* 23, 83-86.

Chandlee, J. M., Scandalios, J. G. (1984). Analysis of variants affecting the catalase developmental program in maize scutellum. *Theoretical and Applied Genetics* 69, 71-77.

Ha, C., Coombs, S., Revill, P. A., Harding, R. M., Vu, M., Dale, J. L. (2008). Design and application of two novel degenerate primer pairs for the detection and complete genomic characterization of potyviruses. *Archives of Virology* 153, 25-36.

Hagel, J. M., Weljie, A. M., Vogel, H. J., Facchini, P. J. (2008). Quantitative 1H Nuclear Magnetic Resonance Metabolite Profiling as a Functional Genomics Platform to Investigate Alkaloid Biosynthesis in Opium Poppy1[W]. *Plant Physiology* 147,1805-1821

Heath, R. L., Packer, L. (1968). Photoperoxidation in isolated chloroplasts. I. Kinetic and stoichiometry of fatty acid peroxidation. *Archives of Biochemistry and Biophysics* 125, 189-198.

Lichtenthaler, H. K., Buschmann, C. (1987). Chlorophylls and carotenoids: Measurement and characterization by UV-VIS Spectroscopy. *Current Protocols in Food Analytical Chemistry* F4.3.1-F4.3.8.

Padidam, M., Beachy, R. N., Fauquet, C. M. (1995). Tomato leaf curl geminivirus from India has a bipartite genome and coat protein is not essential for infectivity. *Journal of General Virology* 76, 25-35.

Rojas, M. R., Gilbertson, R. L., Russell, D. R., Maxwell, D. P. (1993). Use of degenerate primers in the polymerase chain reaction to detect whitefly-transmitted geminiviruses. *Plant Disease* 77, 340-347.

Srivastava, A., Raj, S. K., Kumar, S., Snehi, S. K., Kulshreshtha, A., Hallan, V., Pande, S. S. (2013). Molecular identification of *Ageratum enation virus*, betasatellite and alphasatellite molecules isolated from yellow vein diseased *Amaranthus cruentus* in India. Virus Genes 47, 584-590.

**Figure S1. Symptoms, and virus detection and identification in infected poppy leaf. (a):** Close view of an infected leaf showing vein thickening (enation on the secondary veins) on the abaxial side. **(b):** Gel image showing bands of 1.0 kb and 1.2 kb sizes obtained with degenerate CP and betasatellite primers respectively in 10 randomly selected field samples (Lanes 1-10) corresponding to a positive control (Lane = P). While such bands absent in healthy tissue (Lane = N). Lane M = 1 kb ladder. The degenerate alphasatellite and potyvirus primers could not amplify the expected genes in any of these 10 samples. **(c)&(d):** Phylogenetic analyses of AEV and ALCB using ML method showing their close relationships with isolates of AEV and ALCB respectively. The bar indicates the number of substitutions per nucleotide position. Full names of viruses may be found by reference to the GenBank accessions.

**Figure S2. AEV infection reduces plant biomass and chlorophyll content.** Graphs of growth parameters of AEV infected poppy plants showing reduction in the selected attributes (stem length, stem diameter, length of third leaf from the top, capsule diameter, fresh and dry weight) as compared to the healthy ones. The amount of chlorophyll is also reduced in infected leaves. Data is given as means ± SEM, calculated using six biological replicates.

**Figure S3. Genome organization of AEV and ALCB.** Map of AEV showing the sense (AV1and AV2) and complementary (AC1, AC2, AC3, and AC4) strand ORFs. The IR and viral genes V1, V2, C1, C2, C3 and C4 predicted to express the pre-CP, CP, Rep, transactivator (TrAP), replication enhancer (REn) and silencing suppressor proteins respectively, are shown. Map of ALCB showing the complementary strand ORF, betaC1, encoding beta C1 protein. In addition to this, ALCB has an A-rich region and satellite conserved region (SCR). The drawing of genomic components is not to the scale.

**Figure S4 Infectivity assay.** Severe leaf curl and vein thickening symptoms developed in poppy plants inoculated with agroinfectious cloned DNAs of AEV (PAS-AEV) and ALCB (PAS-ALCB). The representative plant picture of 45 days post inoculation (dpi) and 60 dpi is shown. The close up of infected and healthy leaves and capsules are also shown for symptom comparison.

**Figure S5.** **Alteration in stem and leaf anatomy of virus infected poppy plants**. Microscopic view of CS of healthy (a-d) and infected (e-h) stem and leaf (i-k) of poppy plants. **(a)&(e)**: general view of stem CS showing epidermis, cortex, vascular bundles and pith regions. **(b-d)**: Cortex, vascular bundles and pith cells showing healthy, tightly packed and turgid cells in control plants with differentiated tissues. **(e-h)**: Cortical cells showing detachment from epidermis, phloem is merged with laticifers and increased sclerenchyma in virus infected stem CS. **(i)&(j)**: Healthy vascular bundles and pith cells in mid vein region of leaves in control poppy plants. **(k)&(l)**: altered vascular bundles in leaves showing excess retention of the trypan blue dye showing loss of membrane integrity. Magnification: a and e: 4X; b, f, i and k: 10X; c and g: 40X; d, h, j, l: 100X.

**Figure S6. Virus infection upregulated the production of stress related enzymes.** Defense related antioxidant enzymes LPX**,** proline**,** CAT**,** SOD and APX were significantly upregulated in AEV infected plants. The normal level of these enzymes has been shown in their respective healthy controls.

**Figure S7.** **Chromatogram showing alkaloid contents in infected and healthy opium poppy capsules. (a):** Chromatogram showing morphine, codeine, thebaine, noscapine and papaverine contents, obtained by HPLC, in healthy capsule samples. **(b):** Image showing reduction in alkaloid contents (except noscapine) in infected capsules. **(c):** Standards of morphine (M), codeine (C), thebaine (T), noscapine (N) and papaverine (P) as controls. The alkaloid contents in sample were calibrated in reference to the standard curve.

**Figure S8.** Principal component analysis of metabolites of AEV infected leaves and capsules with comparison to their healthy controls suggesting high metabolite perturbation in AEV infected capsules.

**Table S1.** List of primers used for detection of virus, biosynthetic pathways of opium, and quantification of virus genome.

**Table S2** Infectivity assay on *P. sominiferum*, *N. glutinosa, S. lycopersicon* and *Ageratum conyzoids* plant host species employing cloned agroinfectious DNAs of AEV and ALCB.

**Table S3.** Metabolomic screening of opium poppy leaves and fruits (healthy v/s infected) by GC-MS analysis.

**Figure S1**

**Figure S2**

**Figure S3**

**
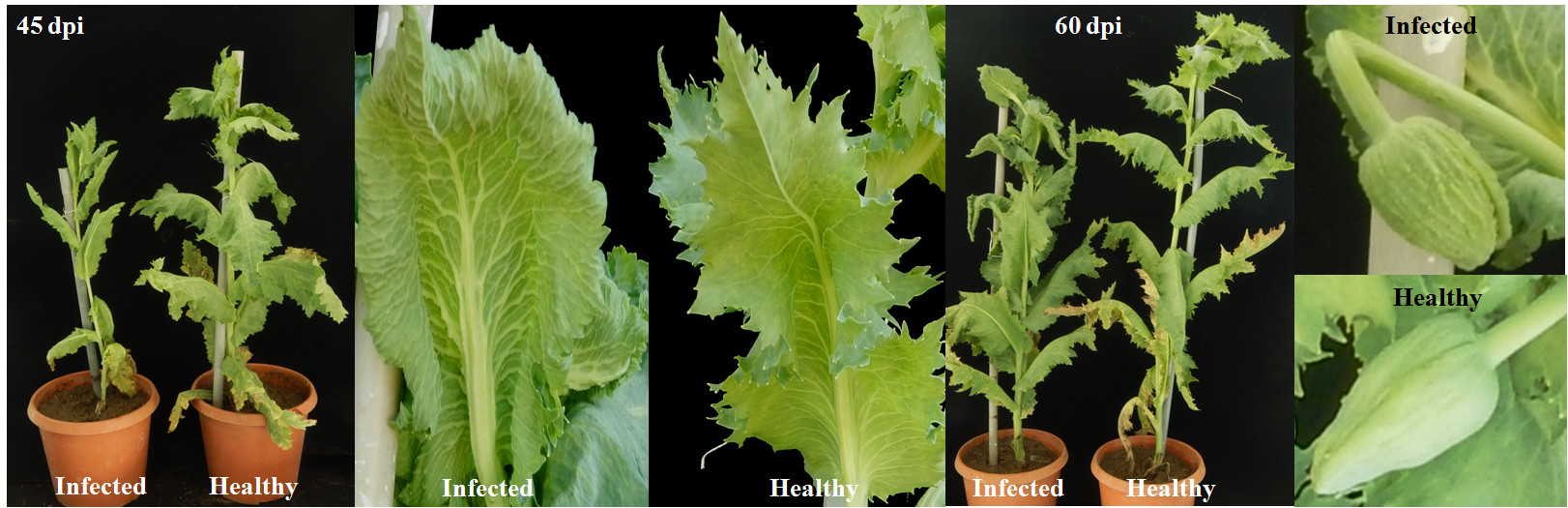
**

**Figure S4**

**Figure S5**

**Figure S6**

**Figure S7**

**Figure S8**

**Table S1**

| **Primer Name** | **Sequence (5’-3’)** | **Primer length (nt)** | **Reference** |
| --- | --- | --- | --- |
| DNAACP-F | ATGGCGAAGCGACCAG | 16 | Rojas *et al*., 1993 |
| DNAACP-R | TTAATTTGTGACCGAATCAT | 20 |
| DNAB-F | GAGTTTCCGYTTGTGGAAGWCCCGGAAGT | 29 | Padidam *et al*., 1995 |
| DNAB-R | YTKGAYTTYGGTCTGTKG | 18 |
| Alpha-F | CTGCAGATAATGTAGCTTACCAG | 23 | Bull *et al*., 2003 |
| Alpha-R | GATATGTGCACCTCCTAGACGTC | 23 |
| Beta-F | AGCCTTAGCTACGCCGGAGC | 20 | Briddon *et al*., 2003 |
| Beta-R | GCTGCGTAGCGTAGAGGTTT | 20 |
| PotyCI-F | GGIVVIGTIGGIWSIGGIAARTCIAC | 26 | Ha *et al*., 2008 |
| PotyCI-R | ACICCRTTYTCDATDATRTTIGTIGC | 26 |
| NCS-F | AGAGATGTTCTGCTTCCTGGTG | 22 | Hagel *et al*., 2008 |
| NCS-R | TCCTCCTTCGATCATCACCACT | 22 |
| CNMT-F | TCGTTGAACATGTTTGCCAT | 20 |
| CNMT-R | GAGTCAGAGTTCCAGCAGGG | 20 |
| 4OMT-F | GACAACAAACTTGTGCTGC | 19 |
| 4OMT-R | CTTGTTTCTCCAGCTTGC | 18 |
| BBE-F | TCCATTGATGTCTTGTCGGA | 20 |
| BBE-R | TACCAACAGTCGGACACCAA | 20 |
| COR-F | TCTGGTGCGCTGATGCTCACG | 21 |
| COR-R | TCTGACACTCTTCCATGGCTGC | 22 |
| CODM-F | TTGTGCTTAAATTTCGTGGATGAC | 24 |
| CODM-R | TGATTACATCACTTGACCCAAACAG | 25 |
| SalSyn-F | ATCCATGCAAGAAAGAGCGT | 20 |
| SalSyn-R | ACTTGCCAACCCACTTATGC | 20 |
| SalAT-F | GATGTGCCACTAAGCCAGCT | 20 |
| SalAT-R | TGTAGCTGCATCGGCAATCT | 20 |
| T6ODM-F | TTGAGGCACAAATGAGAAAATTGA | 24 |
| T6ODM-F | CACAACGCACTTTCGAGAAATTAC | 24 |
| TNMT-F | CCATGTTACAGGGCTCACTAATT | 23 |
| TNMT-R | CAAACGTCGACGCCATACTAG | 21 |
| N7OMT-F | CGACCGTTTGTACCGAGTTATG | 22 |
| N7OMT-R | CAGAGAAGTACTGGTTAGCTCCACC | 25 |
| AEV-CP-F | GTCGTCCTCAGGATACCCCT | 20 |
| AEV-CP-R | CACAGTCGCATGCCATTTCC | 20 |
| β-Actin-F | TCTCAACCCAAAGGCTAATCG | 21 |
| β-Actin-R | CCCCAGAATCCAAGACAATAC | 21 |

CP = coat protein, CI = cylindrical inclusion protein, AEV = ageratum enation virus.

**Table S2**

| **Plant species** | **Agroinfectious clones** | **Symptoms developed** | **Symptoms**  **developed at dpi** | **Number of**  **symptomatic/**  **inoculated plants** | | | ***Detection of begomovirus**  **and betasatellite by PCR** |
| --- | --- | --- | --- | --- | --- | --- | --- |
|  |  |  |  | **Exp-1** | **Exp-2** | **Exp-3** |  |
| *P. sominiferum* | PAS-AEV | No symptom | - | 00/20 | 00/20 | 00/20 | 00/60 |
|  | PAS-AEV+ PAS-ALCB | Severe upward leaf curl, vein thickening, and stunting | 35 | 07/20 | 12/20 | 13/20 | 32/60 |
|  | PAS-ALCB | No symptom | - | 00/20 | 00/20 | 00/20 | 00/60 |
| *N. glutinosa* | PAS-AEV | Mild leaf curl | 30 | 16/20 | 18/20 | 16/20 | 50/60 |
|  | PAS-AEV+ PAS-ALCB | Severe leaf curl, yellowing, vein thickening, and stunting | 25 | 17/20 | 16/20 | 19/20 | 52/60 |
|  | PAS-ALCB | No symptom | - | 00/20 | 00/20 | 00/20 | 00/60 |
| *S. lycopersicon* | PAS-AEV | Mild leaf curl | 30 | 14/20 | 17/20 | 16/20 | 47/60 |
|  | PAS-AEV+ PAS-ALCB | Severe upward leaf curl, growth stunting | 30 | 17/20 | 16/20 | 16/20 | 49/60 |
|  | PAS-ALCB | No symptoms | - | 00/20 | 00/20 | 00/20 | 00/60 |
| *A. conyzoids* | PAS-AEV | Mild vein yellowing | 25 | 17/20 | 18/20 | 14/20 | 49/60 |
|  | PAS-AEV+ PAS-ALCB | Severe leaf curl, yellowing, and stunting | 25 | 18/20 | 17/20 | 17/20 | 52/60 |
|  | PAS-ALCB | No symptom | - | 00/20 | 00/20 | 00/20 | 00/60 |

*Detection of begomovirus and betasatellite was done using their degenerate primers described in MM section.- = data not available.

**Table S3**

| **Rt (min)** | **Metabolites** | **Molecular Formula** | **Fragmentation pattern** | **Concentration** | | | |
| --- | --- | --- | --- | --- | --- | --- | --- |
|  |  |  |  | **Leaf (stdv)** | | **Capsule (stdv)** | |
|  |  |  |  | **Healthy** | **Infected** | **Healthy** | **Infected** |
| 6.05 | Propanoic acid | C_9_H_22_O_3_Si_2_ | m/z234,228,219,147,117,73,66,59 | 1.02±0.13 | 0.96±.12 | 1.82±0.24 | 1.08±0.14 |
| 9.22 | Oxalic acid | C_8_H_18_O_4_Si_2_ | m/z234,220,147,133,100,73,59 | 0.10±0.01 | 0.00±0.00 | 0.00±0.00 | 0.00±0.00 |
| 10.93 | Glycerol | C_12_H_32_O_3_Si_3_ | m/z308,293,218,177,147,133,103,73,59 | 4.88±0.63 | 3.59±0.46 | 6.62±0.86 | 5.99±0.75 |
| 11.96 | GABA | C_10_H_24_O_3_Si_2_ | m/z248,235,233,218,177,75,73,66 | 0.24±0.03 | 0.35±0.05 | 0.09±0.01 | 0.00±0.00 |
| 12.17 | Malonic acid | C_9_H_20_O_4_Si_2_ | m/z248,212,148,147,73,66,61 | 0.08±0.01 | 0.09±0.01 | 0.27±0.04 | 0.01±0.00 |
| 12.49 | Glycine | C_11_H_29_NO_2_Si_3_ | m/z291,262,174,133,100,86,73 | 0.19±0.02 | 0.08±0.01 | 0.00±0.00 | 0.00±0.00 |
| 13.42 | Phosphoric acid | C_9_H_27_O_4_PSi_3_ | m/z314,299,283,193,135,91,73 | 1.62±0.21 | 8.04±0.49 | 7.40±0.94 | 10.05±1.30 |
| 13.77 | Glyceric acid | C_12_H_30_O_4_Si_3_ | m/z322,309,292,205,189,147,103,73 | 12.74±1.65 | 17.96±2.33 | 7.47±0.95 | 1.04±0.04 |
| 15.31 | Succinic acid | C_16_H_38_O_6_Si_4_ | m/z262,249,172,147,83,75,73,55 | 2.46±0.31 | 1.93±0.25 | 1.14±0.15 | 0.26±0.13 |
| 15.41 | Fumaric acid | C_10_H_20_O_4_Si_2_ | m/z 260,247,245,157,143,133,75,73 | 0.09±0.01 | 0.01±0.00 | 0.04±0.01 | 0.01±0.00 |
| 15.87 | Maleic acid | C_10_H_20_O_4_Si_2_ | m/z260,247,245,157,143,133,75,73 | 0.14±0.02 | 0.01±0.00 | 0.27±0.03 | 0.01±0.00 |
| 15.92 | β-Alanine | C_12_H_31_NO_2_Si_3_ | m/z305,290,248,232,174,147133,86,73 | 0.13±0.02 | 0.25±0.02 | 0.17±0.04 | 0.00±0.00 |
| 16.38 | Erythritol | C_16_H_42_O_4_Si_4_ | m/z410,395,218,204,149,117,103,73 | 0.31±0.03 | 0.00±0.00 | 0.51±0.06 | 0.00±0.00 |
| 18.32 | Erythronic acid | C_16_H_40_O_5_Si_4_ | m/z424,379,292,220,130,117,89,73 | 0.63±0.08 | 0.49±0.06 | 0.43±0.05 | 0.00±0.00 |
| 18.62 | Malic acid | C_13_H_30_O_5_Si_3_ | m/z350,335,245,175,147,75,73 | 5.64±0.73 | 2.05±0.48 | 5.53±0.70 | 1.00±0.00 |
| 19.14 | L-Threonic acid | C_16_H_40_O_5_Si_4_ | m/z424,409,319,292,205,147,73 | 2.64±0.32 | 3.12±0.39 | 1.46±0.19 | 0.00±0.00 |
| 19.41 | L-Tartaric acid | C_16_H_38_O_6_Si_4_ | m/z436,423,333,292,219,147,117,73 | 0.00±0.00 | 0.00±0.00 | 0.71±0.09 | 0.00±0.00 |
| 20.56 | Ribitol | C_20_H_52_O_5_Si_5_ | m/z512,442,397,259,205,147,73,71 | 0.37±0.05 | 0.22±0.03 | 0.31±0.04 | 0.00±0.00 |
| 20.66 | Xylitol | C_20_H_52_O_5_Si_5_ | m/z512,422,319,217,205,147,103,73 | 0.34±0.04 | 0.13±0.02 | 0.16±0.02 | 0.00±0.00 |
| 21.84 | 2-Ketogluconic acid | C_21_H_50_O_7_Si_5_ | m/z554,511,421,333,277,217,148,103,73 | 0.28±0.04 | 0.26±0.03 | 0.20±0.03 | 0.00±0.00 |
| 22.11 | L-Proline | C_11_H_23_NO_3_Si_2_ | m/z273,258,174,156,147,117,75,73 | 0.58±0.07 | 1.31±0.04 | 1.02±0.05 | 0.00±0.00 |
| 22.53 | D-Fructopyranose | C_21_H_52_O_6_Si_5_ | m/z540,437,319,292,204,147,129,73,69 | 3.35±0.43 | 1.59±0.20 | 3.36±0.43 | 4.47±0.58 |
| 22.83 | D-Fructofuranose | C_21_H_52_O_6_Si_5_ | m/z540,437,257,217,147,73,72 | 3.77±0.47 | 2.52±0.33 | 1.25±0.15 | 0.21±0.03 |
| 24.51 | D-Fructose | C_22_H_55_NO_6_Si_5_ | m/z569,364,309,277,217,147,103,73 | 10.44±1.35 | 12.51±1.62 | 14.51±1.85 | 16.58±2.15 |
| 24.9 | D-Fructosemethyloxime(syn) | C_22_H_55_NO_6_Si_5_ | m/z569,554,307,277,147,103,73 | 8.83±1.14 | 10.80±1.40 | 10.68±1.36 | 13.27±1.72 |
| 25.15 | D-Glucose | C_22_H_55_NO_6_Si_5_ | m/z569,466,320,229,205,160,147,73 | 7.08±0.92 | 10.59±1.37 | 9.24±1.18 | 11.43±1.48 |
| 25.37 | D-Galactose | C_22_H_55_NO_6_Si_5_ | m/z569,376,319,217,205,160,147,103,73 | 7.28±0.92 | 4.22±0.55 | 3.79±0.46 | 4.21±0.54 |
| 25.72 | Citricacid | C_18_H_40_O_7_Si_4_ | m/z460,363,273,183,147,73 | 3.34±0.43 | 1.75±0.22 | 2.56±0.33 | 0.00±0.00 |
| 26.01 | D-Glucosamine | C_18_H_45_NO_5_Si_4_ | m/z467,452,362,204,132,131,73 | 0.00±0.00 | 0.00±0.00 | 0.55±0.07 | 3.84±0.50 |
| 26.21 | Pentaric acid | C_18_H_40_O_7_Si_4_ | m/z480,465,375,273,245,147,75,73 | 4.09±0.53 | 1.19±0.15 | 1.07±0.13 | 0.38±0.05 |
| 26.4 | D-Gluconic acid | C_24_H_60_O_7_Si_6_ | m/z628,613,435,333,217,147,73 | 3.21±0.42 | 2.54±0.33 | 0.50±0.06 | 8.85±1.15 |
| 26.95 | α-D-Glucopyranose | C_21_H_52_O_6_Si_5_ | m/z540,435,305,217,191,147,73 | 3.72±0.48 | 2.60±0.32 | 1.91±0.22 | 4.32±0.56 |
| 27.73 | Myo-inositol | C_24_H_60_O_6_Si_6_ | m/z612,508,367,305,265,191,129,73 | 5.41±0.70 | 5.33±0.69 | 7.65±0.97 | 13.05±1.69 |
| 28.67 | Galactoseoxime | C_24_H_61_NO_6_Si_6_ | m/z627,568,402,322,319,205,103,73 | 1.02±0.11 | 1.05±0.14 | 0.89±0.11 | 1.20±0.15 |
| 31.14 | D-Gulose | C_21_H_52_O_6_Si_5_ | m/z540,361,217,204,145,75,73 | 0.42±0.05 | 0.13±0.02 | 0.48±0.06 | 0.49±0.06 |
| 32.41 | N-Acetyl-Glucosamine | C_21_H_50_N_2_O_6_Si_4_ | m/z538,374,307,217,84,73 | 0.63±0.08 | 0.69±0.09 | 0.63±0.08 | 0.00±0.00 |
| 32.74 | 2-O-Glycerol-α-D-Galactopyranoside | C_27_H_66_O_8_Si_6_ | m/z686,451,361,337,204,147,73 | 0.69±0.09 | 0.36±0.04 | 0.29±0.04 | 0.16±0.02 |
| 32.99 | Mannitol | C_27_H_71_O_9_PSi_7_ | m/z766,651,477,388,239,217,103,73 | 0.34±0.04 | 0.38±0.05 | 0.25±0.03 | 0.00±0.00 |
| 35.04 | D-Glucuronic acid | C_21_H_50_O_7_Si_5_ | m/z554,505,451,375,292,204,147,73 | 0.24±0.03 | 0.30±0.04 | 0.30±0.03 | 0.00±0.00 |
| 36.95 | Sucrose | C_36_H_86_O_11_Si_8_ | m/z918,464,437,361,319,271,217,169,147,73 | 0.55±0.07 | 0.81±0.05 | 0.16±0.02 | 0.30±0.04 |
| 39.27 | D-Glucitol | C_24_H_62_O_6_Si_6_ | m/z614,422,346,307,205,157,147,103,73 | 0.26±0.03 | 0.21±0.03 | 0.00±0.00 | 0.00±0.00 |
| 41.81 | Adenosine(4TMS) | C_22_H_45_N_5_O_4_Si_4_ | m/z555,540,376,280,245,236,230,73 | 0.34±0.04 | 0.00±0.00 | 0.00±0.00 | 0.00±0.00 |
| 42.27 | Morphine | C_23_H_35_NO_3_Si_2_ | m/z429,414,287,236,146,75,73 | 0.65±0.08 | 0.62±0.08 | 0.50±0.06 | 0.00±0.00 |
| 43.28 | Hydromorphone | C_20_H_27_NO_3_Si | m/z357,287,216,162,124,81,73,59 | 0.00±0.00 | 0.00±0.00 | 0.15±0.02 | 0.00±0.00 |
| 43.91 | D-Glucopyranoside | C_36_H_86_O_11_Si_8_ | m/z918,600,597,451,361,205,129,73 | 0.37±0.05 | 0.19±0.02 | 0.00±0.00 | 0.00±0.00 |
| 47.5 | Maltose | C_36_H_86_O_11_Si_8_ | m/z918,361,331,271,217,191,147,117,81,73 | 0.00±0.00 | 0.00±0.00 | 0.91±0.11 | 0.00±0.00 |
